# A glycolysis-related gene signature predicts prognosis of patients with esophageal adenocarcinoma

**DOI:** 10.18632/aging.104206

**Published:** 2020-11-25

**Authors:** Huafeng Kang, Nan Wang, Xuan Wang, Yu Zhang, Shuai Lin, Guochao Mao, Di Liu, Chengxue Dang, Zhangjian Zhou

**Affiliations:** 1 Department of Oncology, The Second Affiliated Hospital of Xi’an Jiaotong University, Xi’an, China; 2 Department of Surgical Oncology, The First Affiliated Hospital of Xi’an Jiaotong University, Xi’an, China

**Keywords:** esophageal adenocarcinoma, glycolysis, gene signature, prognosis, gene set enrichment analysis

## Abstract

Background: Esophageal adenocarcinoma (EAC) is a growing problem with a rapidly rising incidence and carries a poor prognosis. We aimed to develop a glycolysis-related gene signature to predict the prognostic outcome of patients with EAC.

Results: Five genes (CLDN9, GFPT1, HMMR, RARS and STMN1) were correlated with prognosis of EAC patients. Patients were classified into high-risk and low-risk groups calculated by Cox regression analysis, based on the five gene signature risk score. The five-gene signature was an independent biomarker for prognosis and patients with low risk scores showed better prognosis. Nomogram incorporating the gene signature and clinical prognostic factors was effective in predicting the overall survival.

Conclusion: An innovative identified glycolysis-related gene signature and an effective nomogram reliably predicted the prognosis of EAC patients.

Methods: The Cancer Genome Atlas database was investigated for the gene expression profile of EAC patients. Glycolytic gene sets difference between EAC and normal tissues were identified via Gene set enrichment analysis (GSEA). Univariate and multivariate Cox analysis were utilized to construct a prognostic gene signature. The signature was evaluated by receiver operating characteristic curves and Kaplan–Meier curves. A prognosis model integrating clinical parameters with the gene signature was established with nomogram.

## INTRODUCTION

The incidence of esophageal adenocarcinoma (EAC) has increased in recent decades, especially in the Western world. It is the solid tumor with the fastest increase in cases in the United States in the last 30 years [1]. The prognosis of EAC remains poor because it is usually diagnosed late. Although many efforts have been made to improve prevention, early detection, and treatment, the 5-year survival rate for patients with EAC is still less than 15% [2]. Therefore, promising biomarkers may have potential for identifying patients at a high risk of EAC and for evaluating their prognoses, since the progression status of EAC patients does not reflect prognoses and treatment responses accordingly.

Numerous biomarkers have been developed and used to predict the prognoses of cancer patients [3, 4]. Some of them, such as glypican 3 (GPC3) and HER2/neu, were identified through global and targeted metabolite profiling [5, 6]. However, the prognostic efficiency of single-gene biomarkers is limited. Cancer is partly characterized by reprogrammed energy metabolism [7]. EAC is not only a malignant disease but also an energy metabolism disease. It has been recognized that glycolysis pathways are significantly upregulated in the precursor lesions of EAC, which is known as Barrett's esophagus [8]. Increased glycolysis is a Hallmark of cancer metabolism, yet little is known about this phenotype at malignant stages of progression. Gene set enrichment analysis (GSEA) can detect the overall expression of various genes without requiring extensive experience or a clear differential gene threshold. GSEA reveals general trends in the data. Therefore, this emerging computational technology could be applied to statistically analyze gene expression and biological behavior [9]. When applied to studying the glycolytic process, GSEA could provide better understanding of the underlying mechanism of tumorigenesis and the progression of EAC.

In this study, a new gene signature that effectively predicted the outcome of EAC patients was explored by analyzing data from The Cancer Genome Atlas (TCGA) database. We identified three glycolysis-related gene sets (GO glycolytic process, hallmark glycolysis and reactome glycolysis gene sets) and a five-gene risk model (CLDN9, GFPT1, HMMR, RARS and STMN1) to predict the prognostic outcome of EAC patients. CLDN9 has been found to be related to many human malignancies, such as non-Hodgkin's lymphoma, breast cancer, pituitary oncocytomas, laryngeal carcinoma and endometrial cancer, contributing to disease progression and poor prognosis in patients. Wang et al. revealed that CLDN9 was significantly correlated with overall survival and predicted a poorer prognosis in endometrial cancer patients [10]. GFPT1 is the first enzyme of the hexosamine biosynthetic pathway. It transfers an amino group from glutamine to fructose-6-phosphate to yield glucosamine-6-phosphate, thus providing the precursor for uridine diphosphate N-acetylglucosamine (UDP-GlcNAc) synthesis, which is essential for all mammalian glycosylation biosynthetic pathways. Zhang et al. recently suggested that the lncRNA ELFN1-AS1 facilitates the proliferation, migration and invasion of esophageal cancer in vitro by promoting GFPT1 expression [11]. It was also recognized that HMMR was elevated in some malignancies, such as non-small-cell lung, breast, bladder, prostate, colorectal and ovarian cancers, resulting in aggressive phenotypes, poor prognosis and disease progression. Recently, Zhang et al. confirmed the relationship of HMMR involved in a glycolysis related nine-gene risk signature and lung adenocarcinoma in development [12]. Arginyl-tRNA synthetase (RARS) is one of the nine synthetase components of a multienzyme complex, and it belongs to a family of cytoplasmic aminoacyl-tRNA synthetases [13, 14]. Its fusion with MAD1L1 might contribute to tumorigenesis, cancer stem cell like properties and therapeutic resistance [15]. STMN1 functions as a critical element of regulating microtubule dynamics, which is necessary in the final stage of cell division, and its mutation may lead to uncontrolled cell proliferation [16–18]. STMN1 has been reported to be upregulated in several types of cancer tissues and correlated with tumo r aggressiveness [19, 20]. Reports have suggested that higher expression of STMN1 predicts worse survival in patients with several types of solid tumors, such as head and neck squamous cell carcinoma [21], gallbladder carcinoma [22], esophageal squamous cell cancer [23], lung carcinoma [24, 25], breast cancer [26], and endometrial cancer [27]. Javed Akhtar et al also reported that STMN1 may be a suitable target for future therapeutic strategies in distal esophageal adenocarcinoma. Its overexpression was found to be associated with lymph node metastasis and increased malignancy in distal esophageal adenocarcinoma both in vivo and in vitro [28]. Multiple single genes correlated with glycolysis have been reported as a predictor of EAC prognosis; however, no glycolysis-related gene signature has been constructed. In our study, we initially identified a gene signature (CLDN9, GFPT1, HMMR, RARS and STMN1) involved in glycolysis and then proved the predictive ability of this gene signature for EAC. Remarkably, a glycolysis-related gene signature was identified and could be used to evaluate EAC patients’ prognosis independently. Furthermore, a comprehensive nomogram based on clinical factors and gene signatures was established to predict the prognosis of EAC patients.

## RESULTS

### Development of glycolysis-related genes with GSEA

**Figure 1:**
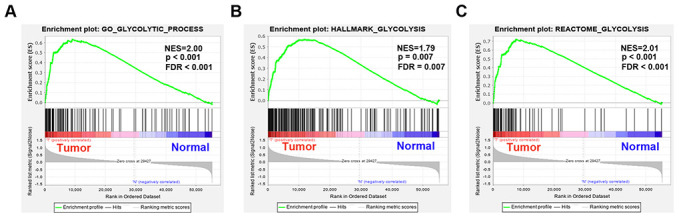
**Enrichment plots between EAC and normal tissues identified by GSEA.** (**A**) GO glycolytic process gene set, (**B**) Hallmark glycolysis gene set, (**C**) Reactome glycolysis gene set. Abbreviations: FDR, False discovery rate; NES, Normalized enrichment score;

**Figure 2:**
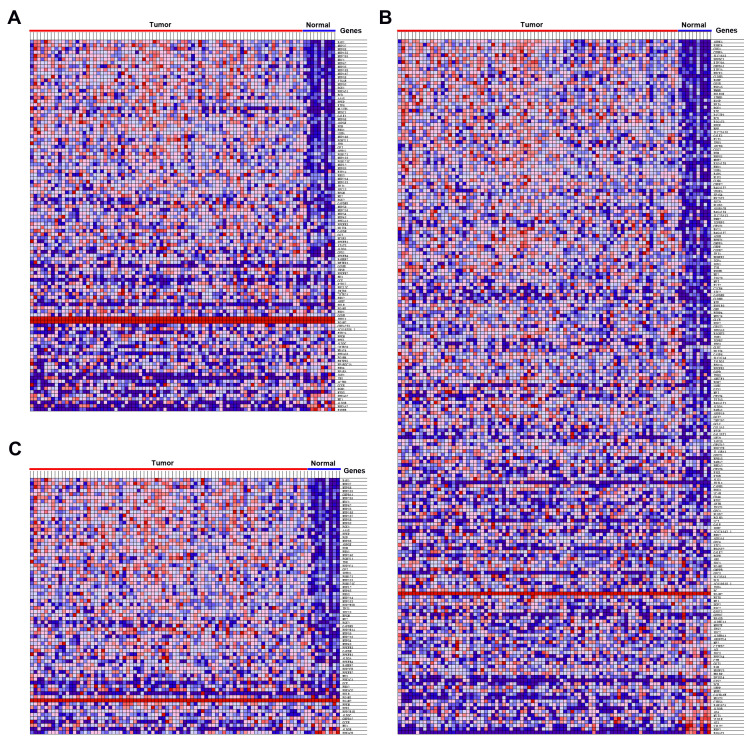
**Heatmap of 378 core genes between EAC and normal tissues.** (**A**) GO glycolytic process gene set, (**B**) Hallmark glycolysis gene set, (**C**) Reactome glycolysis gene set.

TCGA data analysis procedure of this study is shown in Supplementary Figure 1. As shown in the flow chart, GSEA was conducted to identify whether the five glycolysis-related gene sets were significantly different between EAC and normal specimens. The results showed that GO glycolytic process (NES=2.00, normalized P<0.001, FDR < 0.001, Figure 1A), hallmark glycolysis (NES=1.79, normalized P=0.007, FDR=0.007, Figure 1B), and reactome glycolysis (NES = 2.01, normalized P<0.001, FDR<0.001, Figure 1C) gene sets were significantly enriched in cancer samples. However, gene sets from the Biocarta glycolysis pathway (NES=0.93, normalized P=0.615, FDR=0.615) and KEGG glycolysis gluconeogenesis (NES=1.00, normalized P=0.456, FDR=0.456) did not manifest many meaningful results. After screening upregulated gene expression in cancer samples, 106 core genes from the GO glycolytic process gene set (Figure 2A), 200 core genes from the hallmark glycolysis gene set (Figure 2B) and 72 core genes from the reactome glycolysis gene set (Figure 2C) were used in further analysis.

### Expression level of the five-gene signature in EAC and normal tissues

**Figure 3:**
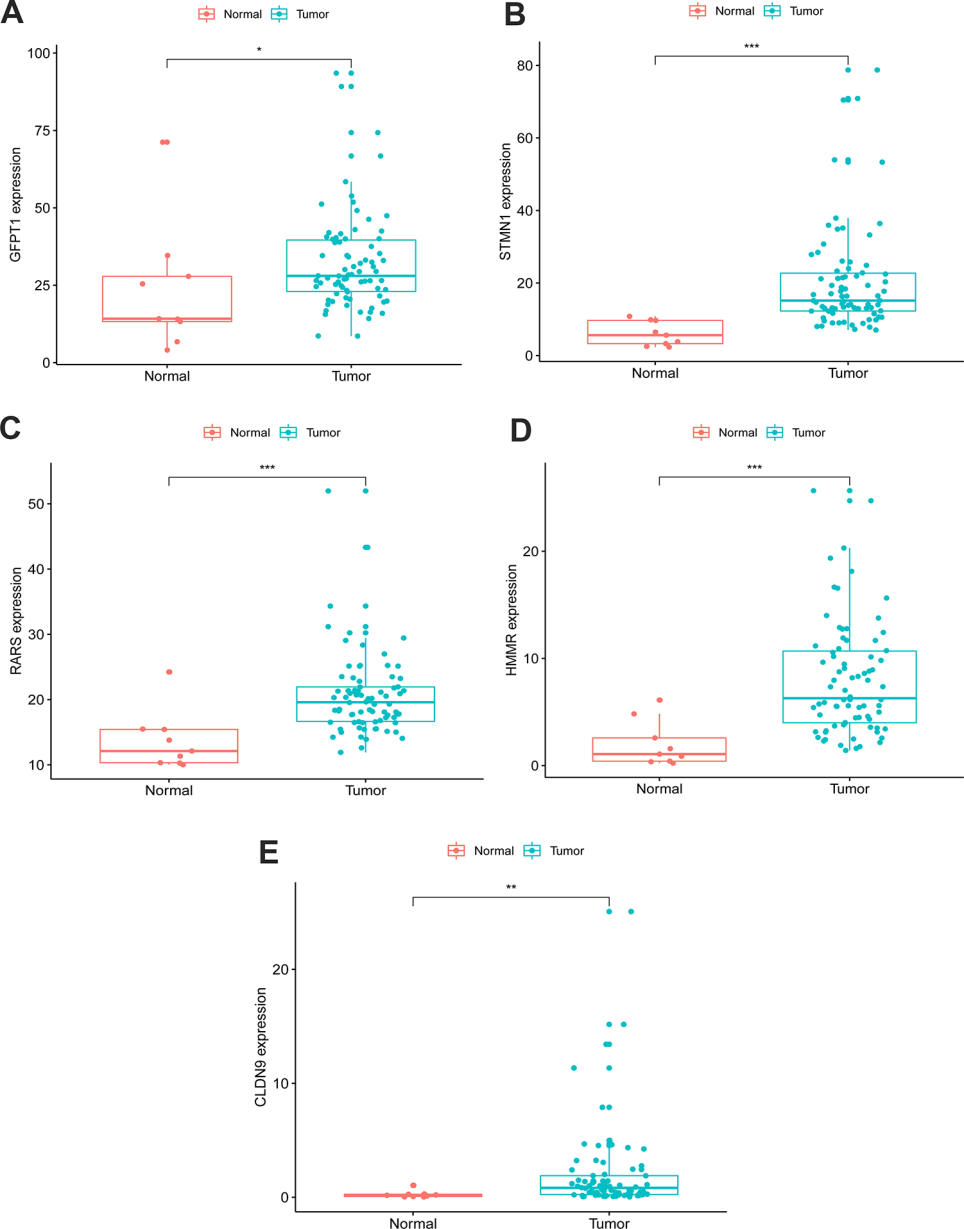
**Expression of the five genes in EAC (n=78) and normal samples (n=9) with unpaired t test.** (**A**) GFPT1, (**B**) STMN1, (**C**) RARS, (**D**) HMMR, (**E**) CLDN9.

An unpaired t test was used to assess the differential expression of 5 genes in 78 EAC tissues and 9 normal tissues. In comparison with normal tissues, results showed that CLDN9, GFPT1, HMMR, RARS and STMN1were upregulated in EAC tissues (Figure 3).

### Mutation status of the five glycolysis-related genes in EAC

**Figure 4:**
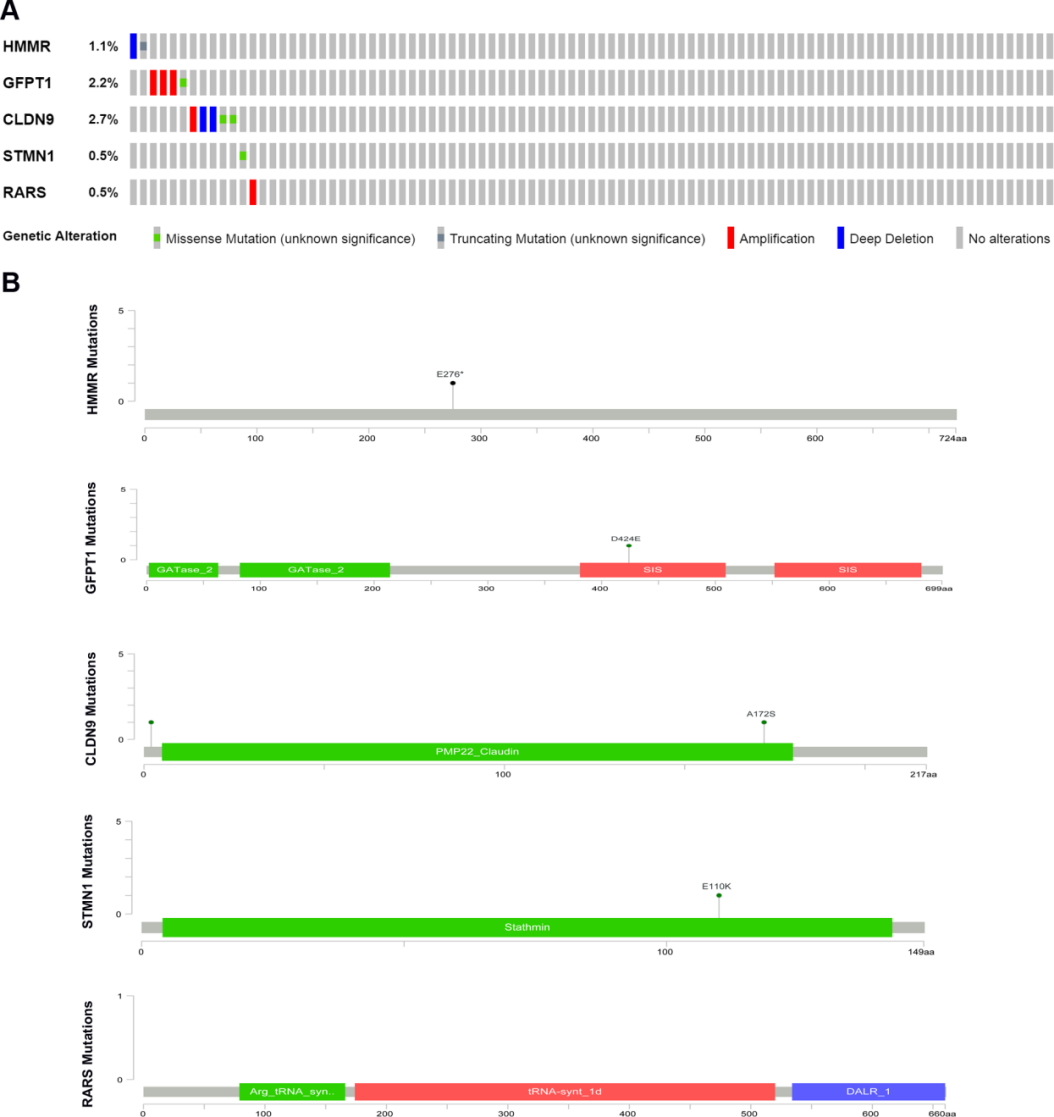
(**A**) The proportion of alteration for the selected genes in 182 clinical samples, (**B**) The five genes’ specific mutation sites.

We then evaluated alterations in the five selected genes by testing 182 EAC samples in the cBioPortal database (https://www.cbioportal.org/). The Results demonstrated that genes on inquiry were altered in 13 (7.14%) of the sequenced cases. The CLDN9 gene was changed in 2.75% of cases, displaying diverse alterations; 1.65% had amplification and 0.55% had deep mutations in GFPT1; The HMMR gene contained one deep deletion sample and one truncating mutation sample; RARS and STMN1 had 0.55% amplification and 0.55% missense mutations, respectively (Figure 4).

### Determination of glycolysis-related genes related to survival of EAC patients

**Figure 5:**
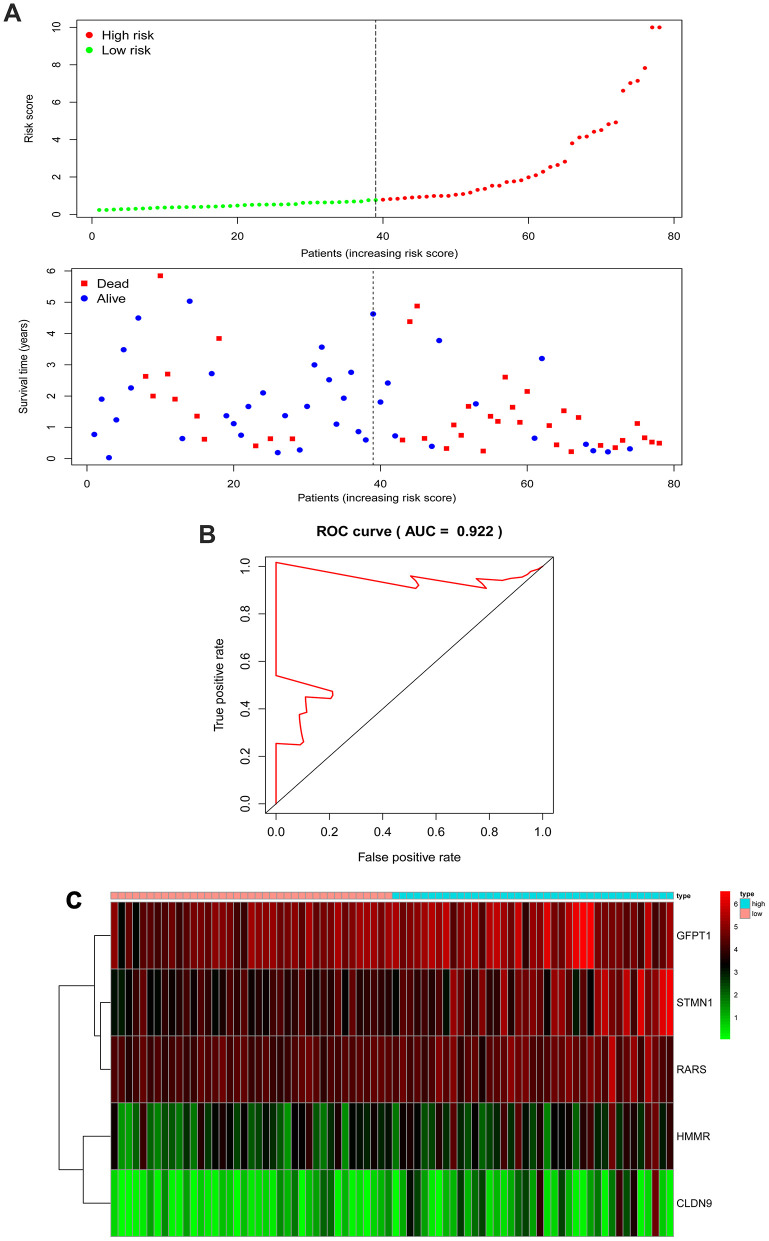
**The five-gene signature associated with risk parameter predicts OS in patients with EAC.** (**A**) The distribution of the five-gene risk score and survival status for each patient. (**B**) ROC curves of the five-gene signature for prediction of 5-year OS. (**C**) A heatmap of five genes’ expression profile. Abbreviations: AUC, areas under the curve; ROC, Receiver operating characteristic.

Univariate Cox hazard analysis was used to identify individual single genes from the three glycolysis-related gene sets that affect the survival of EAC patients, in which we obtained five statistically significant genes: CLDN9, GFPT1, HMMR, RARS and STMN1. The five genes were associated with OS of EAC patients (P < 0.05). Thus, these screened genes were entered into the multivariate regression analysis and were split into a protective role (HMMR) with hazard ratio (HR) < 1 and a risk role (CLDN9, GFPT1, RARS and STMN1) with HR > 1 (Table 1). The joint risk score of the five genes was calculated by substituting the coefficient into the formula to evaluate the prognosis as follows: risk score = (0.1340 × expression of CLDN9) + (0.0347 × expression of GFPT1) + (-0.1031 × expression of HMMR) + (0.0969 ×expression of RARS) + (0.0590 × expression of STMN1). We split patients with EAC in the TCGA cohort into low- and high-risk groups according to the median risk score, based on the five-gene signature. The distribution of survival status and risk score for each patient are displayed in Figure 5A, indicating that patients in the low-risk group had a better survival rate than those in the high-risk group. Additionally, the expression profiles of the five genes are exhibited with a heatmap (Figure 5C). Similarly, ROC curves manifested that the areas under the curve (AUC) at 5-year was 0.922 (Figure 5B), indicating good specificity and sensitivity of the five-gene signature in assessing prognostic outcome for patients with EAC.

**Table 1:** Information of the five prognostic mRNAs associated with overall survival in EAC patients.

**mRNA**	**Ensemble ID**	**location**	**β(cox)**	**HR**	**P**
CLDN9	ENSG00000213937	chr16:3,012,456-3,014,505	0.134	1.143	0.011
GFPT1	ENSG00000198380	chr2:69,319,769-69,387,254	0.035	1.035	0.015
HMMR	ENSG00000072571	chr5:163,460,203-163,491,945	-0.103	0.902	0.006
RARS	ENSG00000113643	chr5:168,486,451-168,519,306	0.097	1.102	0.001
STMN1	ENSG00000117632	chr1:25,884,181-25,906,991	0.059	1.061	0.001

Footnotes: EAC, esophageal adenocarcinoma; chr, chromosome; HR, Hazard ratio.

### The five-gene signature-based risk score acts as an independent prognostic factor

**Figure 6:**
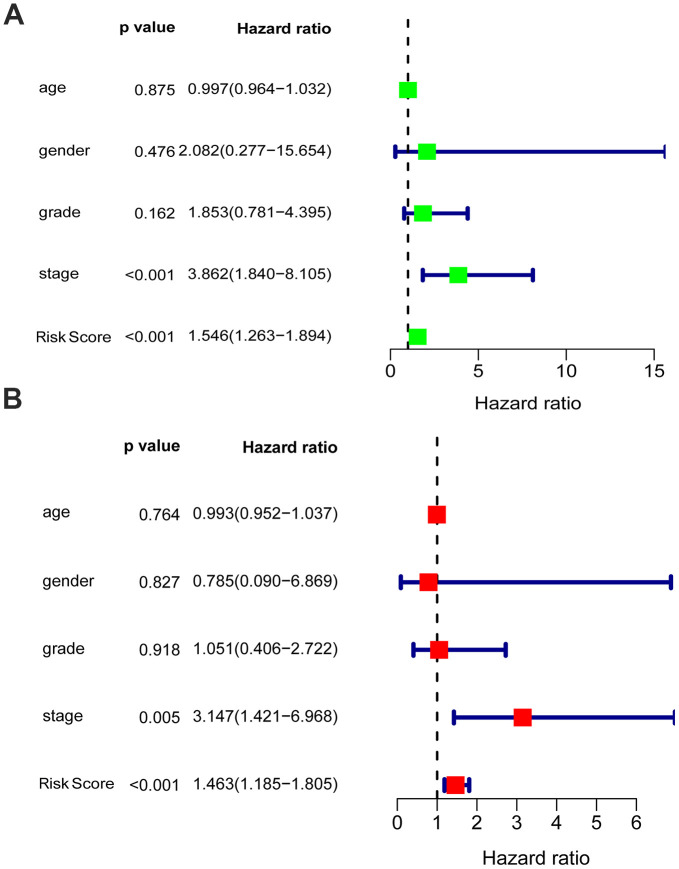
**Univariable and multivariable analyses for the risk score and other clinical characteristics.** (**A**) Univariable analysis, (**B**) Multivariable analysis.

Univariate and multivariate analyses were utilized to analyze the effect of each clinicopathological feature in comparison with the five-gene signature on survival. The prognostic value of the glycolysis-related risk score for OS in EAC patients was tested in combination with clinical features including age, gender, grade and stage. The results of univariate analysis indicated that stage [HR = 3.862, 95% confidence interval (CI): 1.840 - 8.105, P < 0.001] and risk score [HR = 1.546, 95% CI: 1.263 - 1.894, P < 0.001] were correlated with the prognostic outcome (Figure 6A). Subsequently, multivariate Cox analysis suggested that stage [HR = 3.147, 95% CI: 1.421 - 6.968, P = 0.005] and risk score [HR = 1.463, 95% CI: 1.185 - 1.805, P < 0.001] were independent prognostic indices (Figure 6B).

### Validation of the survival predictive ability of the five-gene signature by Kaplan–Meier curve analysis

**Figure 7:**
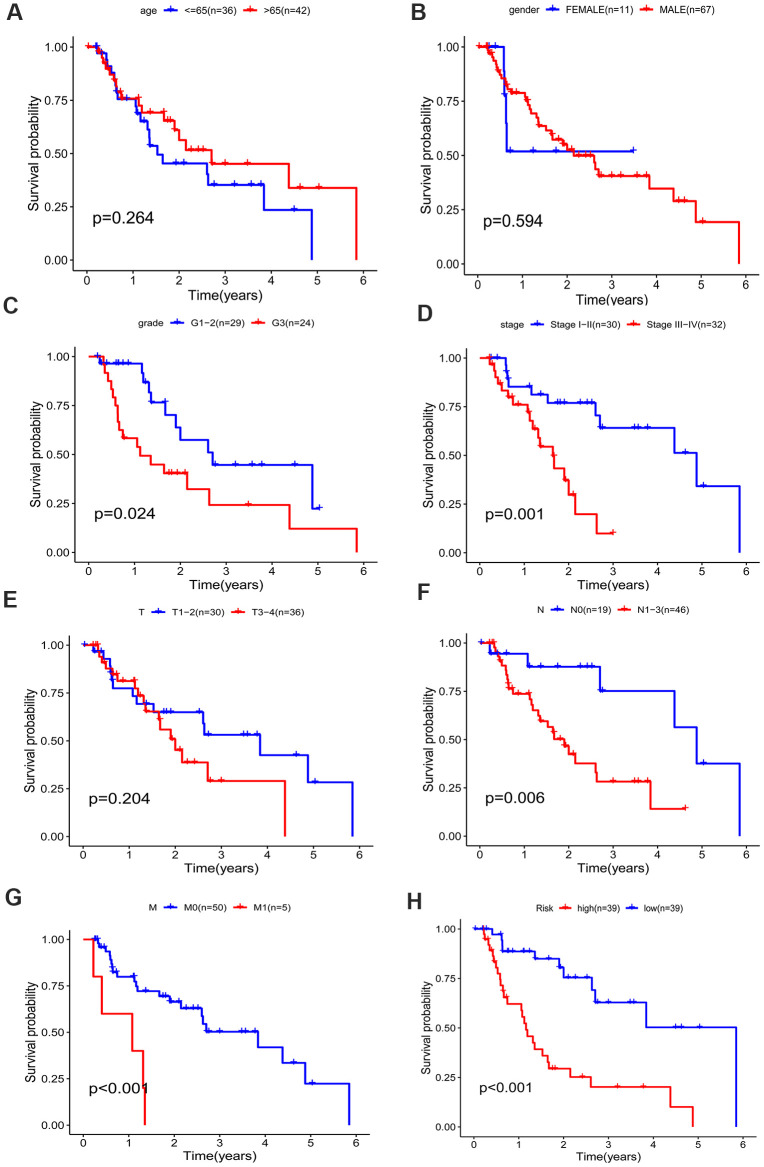
**Kaplan–Meier survival curve of different factors for the patients with EAC in TCGA dataset.** (**A**) age, (**B**) gender, (**C**) grade, (**D**) stage, (**E**) T classification, (**F**) N classification, (**G**) M classification, (**H**) risk.

**Figure 8:**
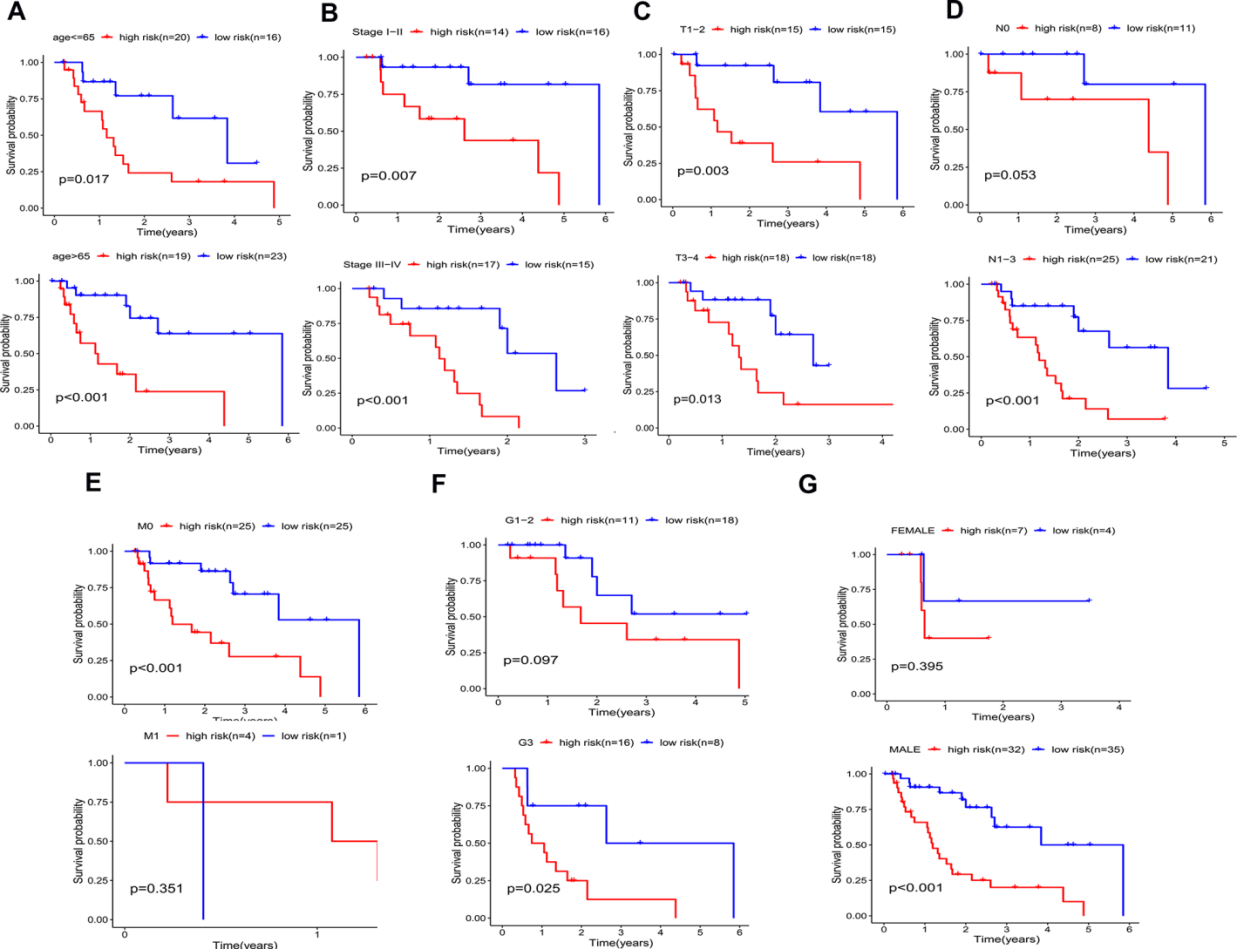
**Kaplan–Meier curves for prognostic value of risk-score signature for the patients divided by each clinical characteristic.** (**A**) age, (**B**) UICC stage, (**C**) T classification. (**D**) N classification, (**E**) M classification, (**F**) Grade, (**G**) gender.

A better prognosis in the low-risk score group was revealed by the log-rank method and Kaplan-Meier survival curves (P < 0.001) (Figure 7H). The UICC (Union for International Cancer Control) stage and five-gene signature were significant in predicting the survival rate of EAC patients in the univariate Cox regression analysis, and the above results were confirmed by the K-M method. Patients at grade 3, III-IV stages (UICC stage) and with lymph node and distant metastasis demonstrated a poor prognosis (Figure 7A–7G), in accordance with the survival curves. Stratified analysis was carried out as the above results further confirmed the accuracy of our analysis. As shown in the K-M curves, the five-gene signature was a dependable prognostic indicator for EAC patients who were in the low-risk group and had a better prognosis (Figure 8A–8C). However, in the N0 (Figure 8D), M1 (Figure 8E), G1-2 (Figure 8F) and female (Figure 8G) subgroups, the risk parameter could no longer independently act as a prognostic marker.

### Construction of a nomogram model integrating the glycolysis-related gene signature

**Figure 9:**
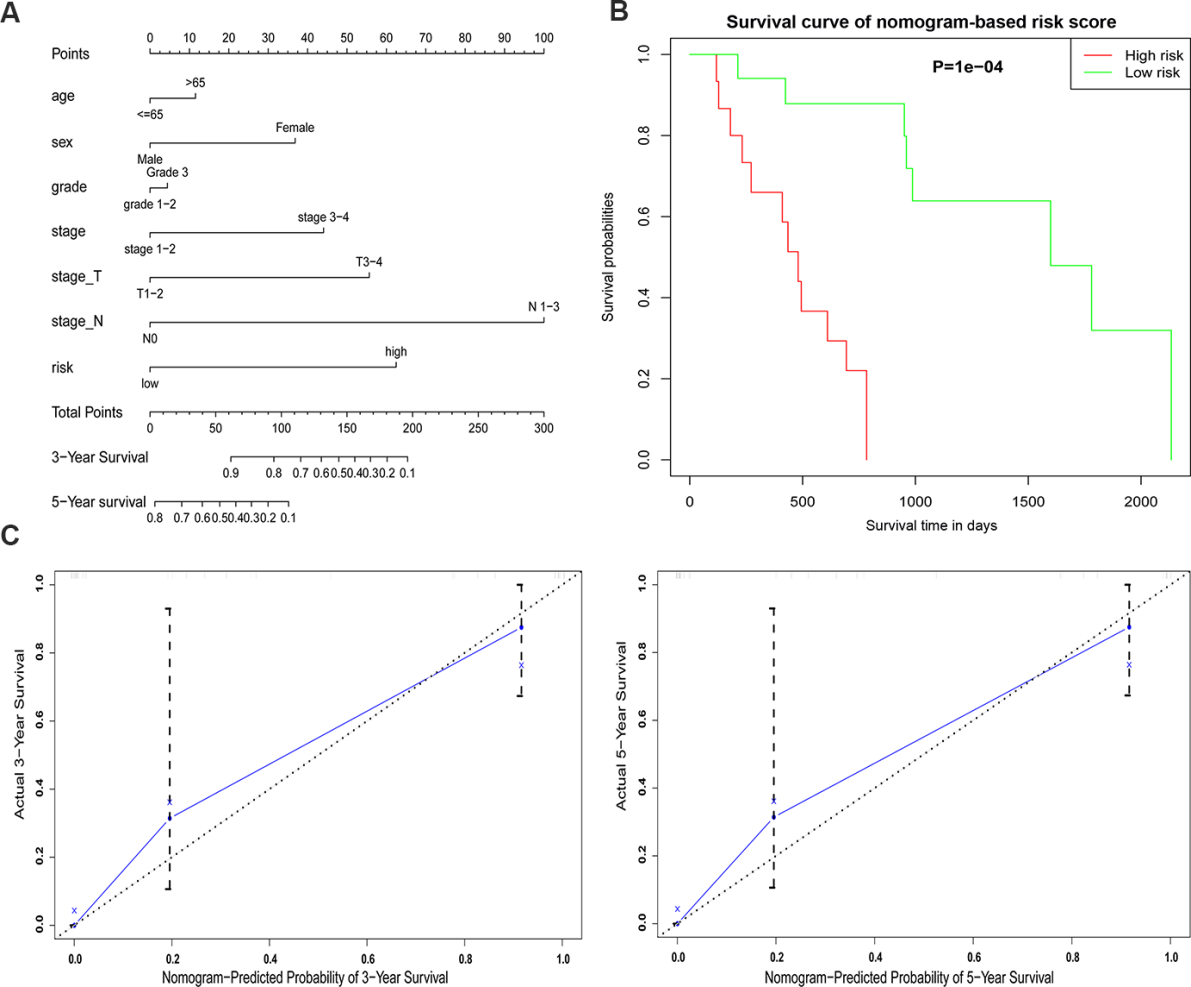
**An established nomogram model predicting 3- and 5-year OS of EAC patients.** (**A**) Nomogram incorporated with the five-gene signature and clinical factors for prediction of the 3- and 5-year OS in patients with EAC in the TCGA dataset. (**B**) Survival curve based on the nomogram grouped by risk score. (**C**) Calibration curve of the nomogram for the prediction of 3- and 5-year OS.

As a tool for use in clinical practice, a nomogram model incorporating the gene signature-based risk score with clinicopathological characteristics (age, gender, grade stage) was constructed to evaluate the survival probability of EAC patients for clinicians (Figure 9A). Survival probability between the two risk groups demonstrated a significant result with P = 0.0001 (Figure 9B). The nomogram demonstrated a worth noting value of the five-gene signature for individualized survival estimation (Figure 9C). The performance of the nomogram was assessed with respect to calibration, discrimination, and clinical usefulness with a C-index of 0.8557.

## DISCUSSION

Increasing studies have verified the significant roles of gender, age, smoking history, pathological stage, tumor size, and lymph node and distant organ metastasis in predicting patient prognosis. Accordingly, more associated mRNAs are molecularly noted to evaluate and predict the prognosis of EAC, suggesting their evaluable clinical significance in studies [29]. For instance, expression of the glycolytic enzyme PKM2 is positively associated with obesity in EAC patients [30], overexpression of uncoupling protein-2 (UCP2) abrogated cigarette smoking condensate and deoxycholic acid mediated increases in lactate and ATP production in EAC; these links may provide novel strategies for EAC therapy [31]. The promising prognostic survival and treatment response based on tumor metabolism and targeting alterations in cellular energetics of EAC patients is in its full swing [32–34]. Studies have examined the prognostic outcomes of EAC patients with cellular glycolysis-related genes. For example, elevated expression of IGF2 mRNA binding protein 2 (IGF2BP2/IMP2) is linked to short survival and metastasis in EAC [35]. Mucin glycoprotein 1 (MUC1) expression increased during progression to EAC and followed tumor invasion [36]. However, these biomarkers were insufficient to independently predict patient prognoses. In particular, multiple factors can affect single gene expression levels. Thus, these biomarkers may be insufficient to be used as prognostic indicators independently and reliably. Therefore, a statistical model made up of genetic markers was used to improve prediction, of which various genes were combined to predict the effect of a single individual gene. A more accurate ability to evaluate the survival outcome of patients with malignancies leads to a widespread application of the model, compared with single biomarkers [37, 38].

Tumors are characterized by unrestricted cell proliferation, which not only eliminates cell cycle control but also gives rise to excessive energy metabolism and eventually results in tumor cell replication and differentiation. Warburg’s landmark observation that cancer cells predominantly convert large amounts of glucose to lactate even under conditions of adequate O_2_ supply is still acknowledged after more than 90 years. Advanced developments in molecular biology and high-throughput molecular analyses have revealed, that the selection for high rates of aerobic glycolysis, which is a prerequisite for unlimited growth, is due to an accumulation of signaling pathways that are altered by gene mutations or changes in gene expression [39]. This suggested that aerobic glycolysis operates in a sophisticated mechanism. Tumor cells proliferate at a speed outpacing the cellular energy supply, thus, redundant nutrient and oxygen consumption by cells could lead to the tumor microenvironment being hypoxic, acidic and lacking of sugar. This phenomenon is more prominent in solid tumors [40]. After nearly a century of unceasing research and exploration, the Warburg effect has been found to take place in various tumors, including breast cancer, lung cancer, gastric carcinoma and colon cancer. Cellular energy metabolic disorders are extensively acknowledged as one of the features of malignancies, although not all tumors demonstrate the Warburg effect. The glycolysis pathway also plays an important role in Barrett’s esophagus developing into EAC, which is illustrated by upregulated pyruvate kinase activity [8]. Thus, a statistical model of glycolysis-related gene signatures comprised of various genes has been built to assess cancer prognosis. Research on large biological datasets has been supported by the technology of the quick development of high-throughput genetic sequencing [19]. A large amount of genomic data was obtained individually to develop new prognostic, diagnostic and immunological biomarkers [20]. Recently, a novel predictive signature was identified by analyzing the gene expression levels or mutations with microarray and RNA-sequencing data. Construction and verification were performed with a Cox proportional hazards regression model [41, 42]. In this study, using bioinformatics methods, we identified genes (CLDN9, GFPT1, HMMR, RARS and STMN1) associated with cellular aerobic glycolysis and exhibited their prognostic ability in EAC. This study facilitates the preceding comprehension of EAC and provides a foundation for further EAC research. We collected glycolysis-related genes and compared data of EAC and normal samples from the EAC dataset in TCGA. We then identified 3 functions displaying significant differences in GSEA. The predictive effect of the five-gene signature for patients with EAC was analyzed with univariate and multivariate Cox regression. Compared with other recognized prognostic estimating indicators, this selected risk profile should be a more efficient classification marker for patients with EAC and a more powerful and targeted prognostic method in evaluating prognostic outcome. We adopted the top-ranking function to screen genes in association with patient survival prediction, instead of wide-range exploration. Kaplan-Meier survival demonstrated that patients with high-risk parameters showed a poor prognosis. The detection and calculation of risk parameters in EAC patients indicated effective clinical value. However, we only made use of OS to predict patient outcomes, due to an inadequate metastasis number and lacking recurrence information from TCGA database, which is one limitation of the study. In addition, the risk parameter predicted EAC patient prognoses in all subgroups except for the female, G1-2, N0, and M1 subgroups, as was displayed in the stratified analysis, though the P value (0.053) of the N0 group infinitely approximated 0.05. The negative result of M1 may be restricted to specimen inadequacy (n=5). The results of the female and G1-2 subgroups indicate that the risk parameter is influenced by the gender and grade of patients with EAC, and the consequence requires deeper investigation. Furthermore, compared with the potential of a single parameter, the combination of clinical characteristics and glycolysis risk score provided a higher potential for clinical application and a more precise prognostic value established with the nomogram.

In conclusion, we identified a glycolysis-related gene signature to predict the prognostic outcome of EAC patients. The five-gene signature was an independent prognostic marker for overall survival, with a lower risk parameter indicating better prognosis. Nomogram integrating clinical factors with this gene signature may not only play a role in predicting EAC patients’ prognosis in clinical practice, but also provides an enlightenment in the underlying mechanisms of cellular glycolysis in tumorigenesis and the identification of more gene targets for EAC treatment.

## MATERIALS AND METHODS

### Data collection

We obtained clinical information and gene expression data of patients with esophageal adenocarcinoma from The Cancer Genome Atlas (TCGA) database. Additionally, the following clinical information was recorded: gender, age, stage, survival status and follow-up time. In total, 78 EAC and 9 normal samples were included for the subsequent study. Detailed information on the overall clinicopathologic features is summarized in Table 2. This study complies with the publication guidelines and access rules of TCGA.

**Table 2:** Clinicopathological parameters of EAC patients.

**Clinical characteristic**	**N**	**%**
Age (years)		
≤65	36	46.15
> 65	42	53.85
Gender		
Male	67	85.90
Female	11	14.10
Grade		
G1-2	29	54.72
G3	24	45.28
T classification		
T1 - T2	30	45.45
T3-T4	36	54.55
N classification		
N0	19	29.23
N1-3	46	70.77
M classification		
M0	50	90.91
M1	5	9.09
UICC stage		
I-II stage	30	48.39
III-IV stage	32	51.61
Survival status		
Alive	40	51.28
Deceased	38	48.72
Mean follow-up time (month)	19.65

Footnotes: EAC, esophageal adenocarcinoma; UICC, Union for International Cancer Control; T, tumor; N, node; M, metastasis.

### Gene set enrichment analysis (GSEA)

To explore whether glycolysis-related genes exhibit statistically significant, concordant differences between EAC and normal samples, five glycolysis-related gene sets, the Hallmark, BioCarta, KEGG, GO and reactome gene sets, were downloaded from the Molecular Signatures Database and analyzed with GSEA software 4.0.3. For selecting gene sets enriched in every phenotype, a normalized enrichment score (NES) was obtained by performing 1000 gene set permutations for each analysis. Finally, subsequent analysis was performed when the false discovery rate (FDR) < 0.1, normalized P < 0.05 and |NES| > 1.6 of the gene set.

### Establishment of the gene signature

To determine core genes that correlate with the prognosis of EAC patients in enriched glycolysis-related gene sets, the mRNA quantification data were matched with the survival status for subsequent analysis. OS-related core genes were identified with the univariate Cox regression analysis (P < 0.05), and subsequent analysis was performed by multivariate Cox regression. A linear joint risk score of gene expression level using regression coefficient β was established. The risk score for each sample was calculated as follows: risk score = (β1 × expression of gene 1) + (β2 × expression of gene 2) + (β3 × expression of gene 3) + (β4 × expression of gene 4) + (β5 × expression of gene 5). The samples were then divided into high- and low-risk groups based on the median risk scores for survival analysis.

### Construction and evaluation of the nomogram

The survival probability of EAC patients was compared by the nomogram model, which integrated with thefive-gene signature with clinicopathologic features; this was performed by R software (version 4.0.2). Calibration plots and C-index were generated as an assessment of the nomogram performance. The clinical outcome prediction is displayed on the y-axis and x-axis separately in the calibration graph, with which an ideal prediction could be indicated with a 45-degree dotted line. Bootstrapping was used as an internal validation to decrease the bias of the C-index’s predictive ability.

### Statistical analysis

Statistical analyses were conducted using Excel software (Microsoft Corporation, California) and R software. The prognostic significance of individual indicators was evaluated by univariate and multivariate Cox proportional hazard regression analyses. Kaplan-Meier curves and log-rank tests were utilized to assess the prognostic outcome. Comparison of the different expression levels between the two groups was performed by unpaired t test. Genetic changes in the 5 glycolysis-related genes in EAC were obtained from the cBioPortal website (http://www.cbioportal.org/). R software (version 4.0.2) was utilized to draw the heatmaps, ROC curves, enrichment, forest and calibration plots. P < 0.05 was regarded as statistically significant.

## Supplementary Material

Supplementary Figures

## References

[1] Lv J, Guo L, Liu JJ, Zhao HP, Zhang J, Wang JH. Alteration of the esophageal microbiota in Barrett’s esophagus and esophageal adenocarcinoma. World J Gastroenterol. 2019; 25:2149–61. 10.3748/wjg.v25.i18.2149 31143067 PMC6526156

[2] Li X, Francies HE, Secrier M, Perner J, Miremadi A, Galeano-Dalmau N, Barendt WJ, Letchford L, Leyden GM, Goffin EK, Barthorpe A, Lightfoot H, Chen E, et al. Organoid cultures recapitulate esophageal adenocarcinoma heterogeneity providing a model for clonality studies and precision therapeutics. Nat Commun. 2018; 9:2983. 10.1038/s41467-018-05190-9 30061675 PMC6065407

[3] Liu S, Miao C, Liu J, Wang CC, Lu XJ. Four differentially methylated gene pairs to predict the prognosis for early stage hepatocellular carcinoma patients. J Cell Physiol. 2018; 233:6583–90. 10.1002/jcp.26256 29115664 PMC13482431

[4] Qixing M, Gaochao D, Wenjie X, Anpeng W, Bing C, Weidong M, Lin X, Feng J. Microarray analyses reveal genes related to progression and prognosis of esophageal squamous cell carcinoma. Oncotarget. 2017; 8:78838–50. 10.18632/oncotarget.20232 29108269 PMC5668002

[5] Salem ME, Puccini A, Xiu J, Raghavan D, Lenz HJ, Korn WM, Shields AF, Philip PA, Marshall JL, Goldberg RM. Comparative molecular analyses of esophageal squamous cell carcinoma, esophageal adenocarcinoma, and gastric adenocarcinoma. Oncologist. 2018; 23:1319–27. 10.1634/theoncologist.2018-0143 29866946 PMC6291329

[6] Rahbari M, Pecqueux M, Aust D, Stephan H, Tiebel O, Chatzigeorgiou A, Tonn T, Baenke F, Rao V, Ziegler N, Greif H, Lin K, Weitz J, et al. Expression of glypican 3 is an independent prognostic biomarker in primary gastro-esophageal adenocarcinoma and corresponding serum exosomes. J Clin Med. 2019; 8:696. 10.3390/jcm8050696 31100935 PMC6572603

[7] Hanahan D, Weinberg RA. Hallmarks of cancer: the next generation. Cell. 2011; 144:646–74. 10.1016/j.cell.2011.02.013 21376230

[8] van Baal JW, Diks SH, Wanders RJ, Rygiel AM, Milano F, Joore J, Bergman JJ, Peppelenbosch MP, Krishnadath KK. Comparison of kinome profiles of Barrett’s esophagus with normal squamous esophagus and normal gastric cardia. Cancer Res. 2006; 66:11605–12. 10.1158/0008-5472.CAN-06-1370 17178854

[9] Thomas MA, Yang L, Carter BJ, Klaper RD. Gene set enrichment analysis of microarray data from pimephales promelas (rafinesque), a non-mammalian model organism. BMC Genomics. 2011; 12:66. 10.1186/1471-2164-12-66 21269471 PMC3037904

[10] Wang ZH, Zhang YZ, Wang YS, Ma XX. Identification of novel cell glycolysis related gene signature predicting survival in patients with endometrial cancer. Cancer Cell Int. 2019; 19:296. 10.1186/s12935-019-1001-0 31807118 PMC6857303

[11] Zhang C, Lian H, Xie L, Yin N, Cui Y. LncRNA ELFN1-AS1 promotes esophageal cancer progression by up-regulating GFPT1 via sponging miR-183-3p. Biol Chem. 2020; 401:1053–61. 10.1515/hsz-2019-0430 32229685

[12] Zhang L, Zhang Z, Yu Z. Identification of a novel glycolysis-related gene signature for predicting metastasis and survival in patients with lung adenocarcinoma. J Transl Med. 2019; 17:423. 10.1186/s12967-019-02173-2 31847905 PMC6916245

[13] Guigou L, Shalak V, Mirande M. The tRNA-interacting factor p43 associates with mammalian arginyl-tRNA synthetase but does not modify its tRNA aminoacylation properties. Biochemistry. 2004; 43:4592–600. 10.1021/bi036150e 15078106

[14] Matsumoto N, Watanabe N, Iibe N, Tatsumi Y, Hattori K, Takeuchi Y, Oizumi H, Ohbuchi K, Torii T, Miyamoto Y, Yamauchi J. Hypomyelinating leukodystrophy-associated mutation of RARS leads it to the lysosome, inhibiting oligodendroglial morphological differentiation. Biochem Biophys Rep. 2019; 20:100705. 10.1016/j.bbrep.2019.100705 31737794 PMC6849085

[15] Zhong Q, Liu ZH, Lin ZR, Hu ZD, Yuan L, Liu YM, Zhou AJ, Xu LH, Hu LJ, Wang ZF, Guan XY, Hao JJ, Lui VW, et al. The RARS-MAD1L1 fusion gene induces cancer stem cell-like properties and therapeutic resistance in nasopharyngeal carcinoma. Clin Cancer Res. 2018; 24:659–73. 10.1158/1078-0432.CCR-17-0352 29133573 PMC5796860

[16] Doye V, Le Gouvello S, Dobransky T, Chneiweiss H, Beretta L, Sobel A. Expression of transfected stathmin cDNA reveals novel phosphorylated forms associated with developmental and functional cell regulation. Biochem J. 1992; 287:549–54. 10.1042/bj2870549 1445213 PMC1133199

[17] Jeon TY, Han ME, Lee YW, Lee YS, Kim GH, Song GA, Hur GY, Kim JY, Kim HJ, Yoon S, Baek SY, Kim BS, Kim JB, Oh SO. Overexpression of stathmin1 in the diffuse type of gastric cancer and its roles in proliferation and migration of gastric cancer cells. Br J Cancer. 2010; 102:710–18. 10.1038/sj.bjc.6605537 20087351 PMC2837578

[18] Bièche I, Lachkar S, Becette V, Cifuentes-Diaz C, Sobel A, Lidereau R, Curmi PA. Overexpression of the stathmin gene in a subset of human breast cancer. Br J Cancer. 1998; 78:701–09. 10.1038/bjc.1998.565 9743287 PMC2062973

[19] Biaoxue R, Hua L, Wenlong G, Shuanying Y. Overexpression of stathmin promotes metastasis and growth of Malignant solid tumors: a systemic review and meta-analysis. Oncotarget. 2016; 7:78994–9007. 10.18632/oncotarget.12982 27806343 PMC5346693

[20] Biaoxue R, Xiguang C, Hua L, Shuanying Y. Stathmin-dependent molecular targeting therapy for Malignant tumor: the latest 5 years’ discoveries and developments. J Transl Med. 2016; 14:279. 10.1186/s12967-016-1000-z 27670291 PMC5037901

[21] Wu H, Deng WW, Yang LL, Zhang WF, Sun ZJ. Expression and phosphorylation of stathmin 1 indicate poor survival in head and neck squamous cell carcinoma and associate with immune suppression. Biomark Med. 2018; 12:759–69. 10.2217/bmm-2017-0443 29847156

[22] Bo X, Wang J, Fu Q, Wang Y, Liu H, Xu J. Stathmin 1 expression predicts prognosis and benefits from adjuvant chemotherapy in patients with gallbladder carcinoma. Oncotarget. 2017; 8:108548–55. 10.18632/oncotarget.19625 29312550 PMC5752463

[23] Ni PZ, He JZ, Wu ZY, Ji X, Chen LQ, Xu XE, Liao LD, Wu JY, Li EM, Xu LY. Overexpression of stathmin 1 correlates with poor prognosis and promotes cell migration and proliferation in oesophageal squamous cell carcinoma. Oncol Rep. 2017; 38:3608–18. 10.3892/or.2017.6039 29039594

[24] Biaoxue R, Hua L, Tian F, Wenlong G. Increased stathmin in serum as a potential tumor marker for lung adenocarcinoma. Jpn J Clin Oncol. 2017; 47:342–49. 10.1093/jjco/hyx005 28158640

[25] Rong B, Nan Y, Liu H, Gao W. Increased stathmin correlates with advanced stage and poor survival of non-small cell lung cancer. Cancer Biomark. 2017; 19:35–43. 10.3233/CBM-160239 28282798 PMC13020710

[26] Kuang XY, Chen L, Zhang ZJ, Liu YR, Zheng YZ, Ling H, Qiao F, Li S, Hu X, Shao ZM. Stathmin and phospho-stathmin protein signature is associated with survival outcomes of breast cancer patients. Oncotarget. 2015; 6:22227–38. 10.18632/oncotarget.4276 26087399 PMC4673159

[27] Reyes HD, Miecznikowski J, Gonzalez-Bosquet J, Devor EJ, Zhang Y, Thiel KW, Samuelson MI, McDonald M, Stephan JM, Hanjani P, Guntupalli S, Tewari KS, Backes F, et al. High stathmin expression is a marker for poor clinical outcome in endometrial cancer: an NRG oncology group/gynecologic oncology group study. Gynecol Oncol. 2017; 146:247–53. 10.1016/j.ygyno.2017.05.017 28532857 PMC5526627

[28] Akhtar J, Wang Z, Yu C, Li CS, Shi YL, Liu HJ. STMN-1 is a potential marker of lymph node metastasis in distal esophageal adenocarcinomas and silencing its expression can reverse Malignant phenotype of tumor cells. BMC Cancer. 2014; 14:28. 10.1186/1471-2407-14-28 24433541 PMC3898730

[29] Dong Z, Wang J, Zhan T, Xu S. Identification of prognostic risk factors for esophageal adenocarcinoma using bioinformatics analysis. Onco Targets Ther. 2018; 11:4327–37. 10.2147/OTT.S156716 30100738 PMC6065599

[30] Lynam-Lennon N, Connaughton R, Carr E, Mongan AM, O’Farrell NJ, Porter RK, Brennan L, Pidgeon GP, Lysaght J, Reynolds JV, O’Sullivan J. Excess visceral adiposity induces alterations in mitochondrial function and energy metabolism in esophageal adenocarcinoma. BMC Cancer. 2014; 14:907. 10.1186/1471-2407-14-907 25471892 PMC4265417

[31] Xu Y, Feingold PL, Surman DR, Brown K, Xi S, Davis JL, Hernandez J, Schrump DS, Ripley RT. Bile acid and cigarette smoke enhance the aggressive phenotype of esophageal adenocarcinoma cells by downregulation of the mitochondrial uncoupling protein-2. Oncotarget. 2017; 8:101057–71. 10.18632/oncotarget.22380 29254145 PMC5731855

[32] Harada K, Wu CC, Wang X, Mizrak Kaya D, Amlashi FG, Iwatsuki M, Blum Murphy MA, Maru DM, Weston B, Lee JH, Rogers JE, Thomas I, Shanbhag N, et al. Total lesion glycolysis assessment identifies a patient fraction with a high cure rate among esophageal adenocarcinoma patients treated with definitive chemoradiation. Ann Surg. 2019. [Epub ahead of print]. 10.1097/SLA.0000000000003228 32675544

[33] Vona-Davis L, Vincent T, Zulfiqar S, Jackson B, Riggs D, McFadden DW. Proteomic analysis of SEG-1 human Barrett’s-associated esophageal adenocarcinoma cells treated with keyhole limpet hemocyanin. J Gastrointest Surg. 2004; 8:1018–23. 10.1016/j.gassur.2004.08.014 15585389

[34] Roedl JB, Colen RR, Holalkere NS, Fischman AJ, Choi NC, Blake MA. Adenocarcinomas of the esophagus: response to chemoradiotherapy is associated with decrease of metabolic tumor volume as measured on PET-CT. Comparison to histopathologic and clinical response evaluation. Radiother Oncol. 2008; 89:278–86. 10.1016/j.radonc.2008.06.014 18701180

[35] Barghash A, Golob-Schwarzl N, Helms V, Haybaeck J, Kessler SM. Elevated expression of the IGF2 mRNA binding protein 2 (IGF2BP2/IMP2) is linked to short survival and metastasis in esophageal adenocarcinoma. Oncotarget. 2016; 7:49743–50. 10.18632/oncotarget.10439 27391348 PMC5226544

[36] Adil Butt M, Pye H, Haidry RJ, Oukrif D, Khan SU, Puccio I, Gandy M, Reinert HW, Bloom E, Rashid M, Yahioglu G, Deonarain MP, Hamoudi R, et al. Upregulation of mucin glycoprotein MUC1 in the progression to esophageal adenocarcinoma and therapeutic potential with a targeted photoactive antibody-drug conjugate. Oncotarget. 2017; 8:25080–96. 10.18632/oncotarget.15340 28212575 PMC5421911

[37] Bao ZS, Li MY, Wang JY, Zhang CB, Wang HJ, Yan W, Liu YW, Zhang W, Chen L, Jiang T. Prognostic value of a nine-gene signature in glioma patients based on mRNA expression profiling. CNS Neurosci Ther. 2014; 20:112–18. 10.1111/cns.12171 24279471 PMC6493176

[38] Cheng W, Ren X, Cai J, Zhang C, Li M, Wang K, Liu Y, Han S, Wu A. A five-miRNA signature with prognostic and predictive value for MGMT promoter-methylated glioblastoma patients. Oncotarget. 2015; 6:29285–95. 10.18632/oncotarget.4978 26320189 PMC4745726

[39] Cairns RA, Harris I, McCracken S, Mak TW. Cancer cell metabolism. Cold Spring Harb Symp Quant Biol. 2011; 76:299–311. 10.1101/sqb.2011.76.012856 22156302

[40] Ganapathy-Kanniappan S, Geschwind JF. Tumor glycolysis as a target for cancer therapy: progress and prospects. Mol Cancer. 2013; 12:152. 10.1186/1476-4598-12-152 24298908 PMC4223729

[41] Shen S, Bai J, Wei Y, Wang G, Li Q, Zhang R, Duan W, Yang S, Du M, Zhao Y, Christiani DC, Chen F. A seven-gene prognostic signature for rapid determination of head and neck squamous cell carcinoma survival. Oncol Rep. 2017; 38:3403–11. 10.3892/or.2017.6057 29130107 PMC5783586

[42] Zhao Y, Varn FS, Cai G, Xiao F, Amos CI, Cheng C. A P53-deficiency gene signature predicts recurrence risk of patients with early-stage lung adenocarcinoma. Cancer Epidemiol Biomarkers Prev. 2018; 27:86–95. 10.1158/1055-9965.EPI-17-0478 29141854 PMC5839302

