# Supplementary material for: A glycolysis-related gene signature predicts prognosis of patients with esophageal adenocarcinoma
Source: Aging (Albany NY). 2020 Nov 25;12(24):25828–44. doi: 10.18632/aging.104206 (PMC7803571; doi:10.18632/aging.104206)
Supplement: Supplementary Figures [file aging-12-104206-s001.pdf]

SUPPLEMENTARY FIGURE

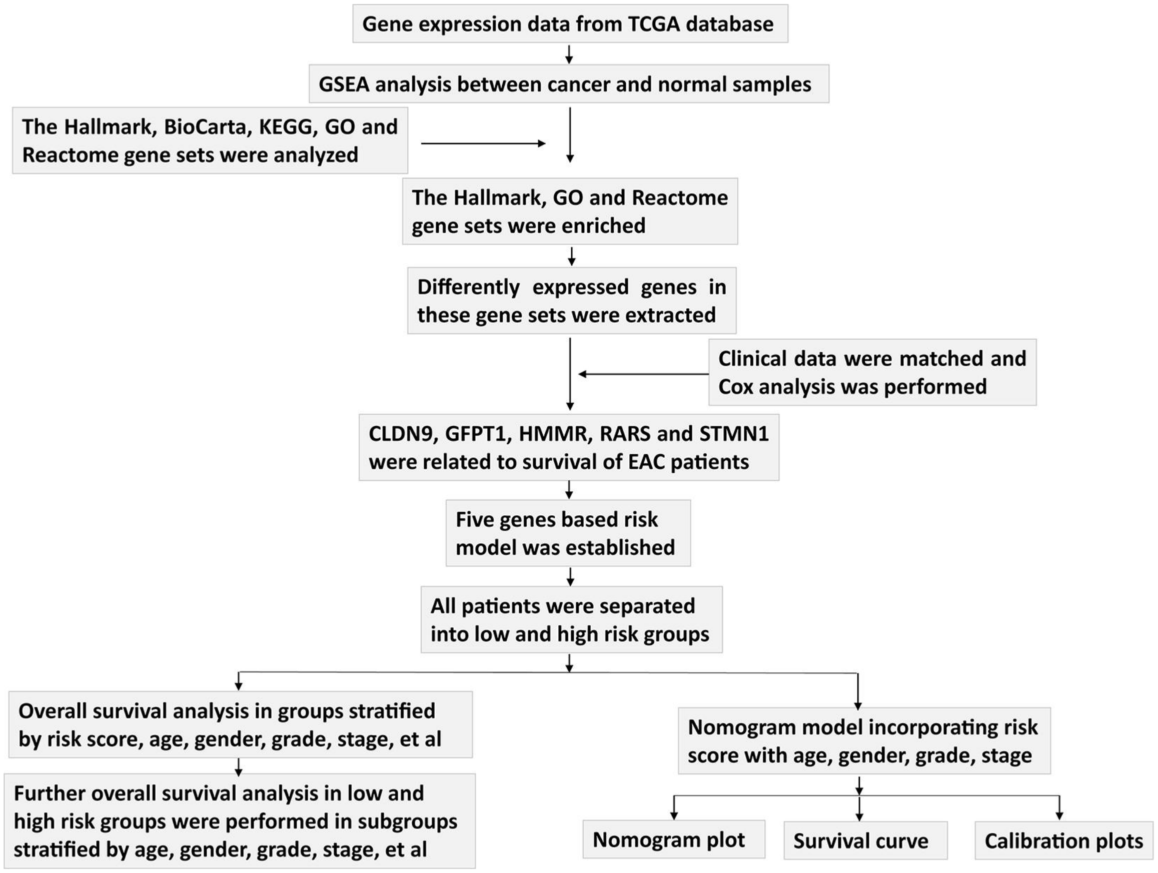

Supplementary Figure 1. Flow chart of the analysis procedure in this study.
